# *Diabetes mellitus* correlates with increased biological age as indicated by clinical biomarkers

**DOI:** 10.1007/s11357-021-00469-0

**Published:** 2021-11-12

**Authors:** Nadine Bahour, Briana Cortez, Hui Pan, Hetal Shah, Alessandro Doria, Cristina Aguayo-Mazzucato

**Affiliations:** 1 Joslin Diabetes Center, Harvard Medical School, 1 Joslin Place, Boston, MA 02215 USA; 2 University of Texas Rio Grande Valley School of Medicine, Edinburg, TX 78539 USA

**Keywords:** Biological age, Chronological age, Diabetes

## Abstract

Chronological age (CA) is determined by time of birth, whereas biological age (BA) is based on changes on a cellular level and strongly correlates with morbidity, mortality, and longevity. Type 2 diabetes (T2D) associates with increased morbidity and mortality; thus, we hypothesized that BA would be increased and calculated it from biomarkers collected at routine clinical visits. Deidentified data was obtained from three cohorts of patients (20–80 years old)—T2D, type 1 diabetes (T1D), and prediabetes—and compared to gender- and age-matched non-diabetics. Eight clinical biomarkers that correlated with CA in people without diabetes were used to calculate BA using the Klemera and Doubal method 1 (KDM1) and multiple linear regression (MLR). The phenotypic age (PhAge) formula was used with its predetermined biomarkers. BA of people with T2D was, on average, 12.02 years higher than people without diabetes (*p* < 0.0001), while BA in T1D was 16.32 years higher (*p* < 0.0001). Results were corroborated using MLR and PhAge. The biomarkers with the strongest correlation to increased BA in T2D using KDM were A1c (*R*^2^ = 0.23, *p* < 0.0001) and systolic blood pressure (*R*^2^ = 0.21, *p* < 0.0001). BMI had a positive correlation to BA in non-diabetes subjects but disappeared in those with diabetes. Mortality data using the ACCORD trial was used to validate our results and showed a significant correlation between higher BA and decreased survival. In conclusion, BA is increased in people with diabetes, irrespective of pathophysiology, and to a lesser extent in prediabetes.

**Supplementary Information:**

The online version contains supplementary material available at 10.1007/s11357-021-00469-0.

## Introduction

The rate of aging varies among species as evidenced by variation up to 100-fold in lifespan among mammals [1]. Members of the same species also vary in the rate of aging, which correlates with their susceptibility to disease, impairment, and death [2]. Hence, individuals of identical chronological age (CA), defined as years lived since birth, can have significant variations in their biological age (BA), the age indicative of the body’s rate of cellular decline or physiological breakdown. Variations in the rate of BA have been shown to be a reliable predictor of mortality, performing significantly better than CA [2].

The concept of BA was first introduced in 1969 as an effort to understand variables that affect mortality and the aging process [3] and refers to quantifiable changes on a cellular level analyzed through biomarkers that can determine specific effects and intensities of disease. According to the National Institutes of Health’s biomarkers definitions, a biomarker is a characteristic that is objectively measured and evaluated as an indicator of normal biological, pathogenic, or pharmacological responses [4]. However, given the complex nature of aging and its associated pathologies, there is not a single biomarker that can be used to calculate BA accurately. Instead, a battery of biomarkers that correlate with aging have shown to be useful in calculating BA [2, 5]. Epigenetic markers and DNA methylation levels are considered the gold standard to calculate BA [6–8]. However, these methods have proven to be expensive and time-consuming, impossible to perform routinely in large populations. Thus, the ability to calculate BA from routinely collected clinical biomarkers provides a powerful tool to predict and monitor health span as well as age-related illnesses such as cardiovascular disease [9], cancer [10], neurodegenerative diseases [11], and type 2 diabetes (T2D).

Over 30 million people are diagnosed with T2D in the USA, most of whom are over 60 years old. Their risk for mortality is 50% higher, and life expectancy is approximately 5 years shorter among men and 7 years shorter among women who have T2D compared to those who do not [12, 13]. Additionally, T2D correlates with significant morbidity and increased risk of serious health complications which further impair health span and lifespan in this population such as blindness, kidney failure, heart disease, stroke, and amputations.

Understanding the correlation between diabetes and BA is important. The current pandemic caused by SARS-CoV-2 further emphasizes the added burden that patients with both type 1 diabetes (T1D) and T2D face. In this unique situation, COVID-19 severity is tripled in the diabetes community [14] where advanced age is one of the main risk factors for complications and death further suggesting a correlation between BA and T2D.

Given the increased morbidity and mortality associated with T2D in a variety of settings, we hypothesized that BA would be increased in individuals with a T2D diagnosis and this would be reflected using mathematical models that employ clinically available biomarkers. We used six different cohorts: patients with T1D and T2D from Joslin Diabetes Center, cases with diabetes, prediabetes, or without a diabetes diagnosis from the National Health and Nutrition Examination Survey 2017–2018 (NHANES), and T2D cases who were recruited in the Action to Control Cardiovascular Risk in Diabetes (ACCORD) Trial. BA was calculated using the Klemera and Doubal method 1 (KDM1) due to its high predictive value [2] and phenotypic age (PhAge), due to its strong correlation with morbidity and mortality [15]. Results were corroborated using multiple linear regression (MLR) and validated using long-term mortality data.

Herein, we show that 8 biomarkers significantly correlated with CA in the control population and were used to calculate BA in groups with T2D, prediabetes, T1D. Seven biomarkers (due to the absence of blood urea nitrogen) were used to calculate BA in the ACCORD trial at the time of recruitment. BA of individuals with T2D, T1D, and a NHANES diabetes cohort was significantly increased. The prediabetes cohort also showed an increase but to a lesser extent. Additionally, we calculated the age ratio, defined as BA/CA, as a surrogate marker of the rate of aging which showed that A1c and systolic blood pressure had the strongest predictive value of age ratio in T2D. Furthermore, we found that BA significantly correlated with mortality risk in longitudinal data from the ACCORD trial validating our results. We conclude that *Diabetes mellitus* (both type 1 and type 2) significantly increased BA and correlated with long-term mortality risk.

## Methods

### Study population

This study was approved by the Joslin Diabetes Center’s Committee on Human Studies (CHS) which determined that it represents human subject research exempt human subject research under 45 CFR 46.104 (d)(4)(ii): it involves the secondary research use of previously collected identifiable private information which was recorded in such a manner that the identity of the human subjects cannot be ascertained directly or through identifiers.

Deidentified clinical data was obtained from male and female subjects, between the ages of 20 and 80 years of age from five different groups: T2D (*n* = 686) and T1D (*n* = 540) from the Joslin Diabetes Center Clinic; diabetes (which does not distinguish between T1 and T2) (*n* = 284), prediabetes (*n* = 76), and people without diabetes (*n* = 873) from NHANES 2017–2018. The NHANES population is described as a noninstitutionalized civilian resident population of the USA [16]. The study includes their socioeconomic status, ethnicity, private health interview, lab tests, and routine physical exams. Every diabetes and prediabetes case was matched by age and gender with a person without diabetes. In the selection of the non-diabetes group, only cases with A1c levels lower than 5.7% were included, such that non-diagnosed prediabetes cases were excluded, as defined by the American Diabetes Association [17]. Additional exclusion criteria included (1) missing clinical values and (2) subject without gender and CA information. Other chronic diseases were not used in the exclusion criteria because in some cohorts the information was not readily available and we wanted a representation of subjects with diabetes that represented comorbidities that are usually present.

The Action to Control Cardiovascular Risk in Diabetes (ACCORD) Trial followed 10,251 T2D adults, ages greater than 40, for 4–8 years with a mean of 5.6 years [18, 19]. Deidentified clinical data was obtained from participants at baseline and correlated with mortality in the follow-up data. Those participants with incomplete clinical data for the 7 biomarkers used in the KDM were excluded.

### Selection of biomarkers

The selection of clinical biomarkers to calculate BA was based in biomarkers obtained from individuals without a diagnosis of prediabetes or diabetes and with an A1c < 5.7%. The aim was to find biomarkers that correlated with CA and could be used to calculate BA. For each clinically available biomarker, we performed Box–Cox transformation to achieve normal distribution followed by standardization. Box–Cox transformation could not be applied to diastolic blood pressure in the NHANES cohort because one individual had a zero value, but it was already approximately normally distributed. We assessed whether biomarkers were redundant by examining the correlations between biomarkers, but no correlation coefficient exceeded 0.75 in absolute value; therefore, no biomarkers were removed. Outlying observations were shrunk through winsorization (i.e., shrinking outlying observations of biomarkers to the border of the main part of the data) and adequate biomarkers were selected using simple univariate linear regression on gender-adjusted data of non-diabetic subjects.

A total of eight biomarkers were selected based on this criterion (Table 1) to analyze T2D, T1D, and diabetes in the Joslin and NHANES 2017–2018 cohorts: creatinine (mg/dL), Serum Albumin (g/dL), cholesterol (mg/dL), urea nitrogen (mg/dL), systolic blood pressure (mmHg), diastolic blood pressure (mmHg), pulse (per min), and A1c (%). Additional analysis substituted A1c by C-reactive protein (CRP) since diabetes inherently affects the former. CRP was used as it has a strong correlation with BA [2] and is frequently used in other models [20].

**Table 1:** Biomarkers that correlated with CA in non-diabetic individuals. These biomarkers were used to calculate BA in T1D and T2D from the Joslin Cohort

Biomarker	*p* value	*R*^2^ value
Creatinine (mg/dL)	7.0 × 10^–3^	0.36
Systolic blood pressure (mmHg)	1.3 × 10^–9^	0.26
Blood urea nitrogen (mg/dL)	3.9 × 10^–3^	0.05
Albumin (g/dL)	1.1 × 10^–2^	0.06
A1c (%)	2.0 × 10^–5^	0.03
Cholesterol (mg/dL)	1.2 × 10^–10^	0.07
Pulse/min	1.6 × 10^–2^	0.03
Diastolic blood pressure (mmHg)	6.6 × 10^–4^	0.03

Due to the absence of blood urea nitrogen in the ACCORD Trial, a total of seven biomarkers were selected based on this criteria (Table 1) to analyze T2D: creatinine (mg/dL), albumin (g/dL), cholesterol (mg/dL), systolic blood pressure (mmHg), diastolic blood pressure (mmHg), heart rate (per min), and A1c (%).

The PhAge model requires that 9 biomarkers are to be included: glucose (mmol/L), albumin (g/L), creatinine (umol/L), red cell distribution width (%), white blood cell count (1000 cells/L), alkaline phosphate (U/L), C-reactive protein (mg/dL), lymphocyte percent (%), and mean cell volume (fL). Due to the absence of biomarkers in the ACCORD and Joslin Cohorts, only the NHANES cohort was used for this analysis.

### Klemera and Doubal method 1 (KDM1)

BA was calculated using KDM1 [2, 21] (INSERT REF 21) using the selected 8 biomarkers by multiple linear regression in control males and females separately. Two thirds of the NHANES non-diabetic control subjects were used to train the KDM algorithm and independent subjects were used as controls (Suppl. Tables 1 and 2). The KDM1 is based on 4 presumptions:

1. Speed of aging is different among species and individuals;
2. BA = CA + RAB (0; S2AB); 
3. Biomarkers used must significantly correlate with CA;
4. X = FX (BA) + RX (0; S2X). Where RAB (0; S2AB), RX (0; S2x) are random variables with zero mean and variance S2AB , S2x respectively, and Fx(BA) is the governing function of a biomarker by BA Detailed steps of KDM were carried out by computer programming by entering the data of indicators such that the model was generated. KDM was run using the R package biomed at https://github.com/bjb40/bioage.

### Multiple linear regression (MLR)

We fit biological age parameters using the 8 biomarkers by multiple linear regression in CTRL males and females separately. We then use training data to calculate out-of-sample biological ages in diabetic (T1D and T2D) males and females separately.

Using the MLR model, aging biomarkers are determined based on their correlation with CA [22].

$${BA}_{i}={b}_{0}+{\sum }_{j=1}^{m}{b}_{j}{x}_{ji}$$

### Phenotypic Age (PhAge)

Phenotypic age was calculated using [23], in which used 9 biomarkers (albumin, glucose, C-reactive protein, lymphocyte percent, mean cell volume, red blood cell distribution width, alkaline phosphatase, and white blood cell count). The equation developed by Levine et al. to calculate phenotypic age is as follows:

$$PhAge=141.50+ \frac{\mathrm{ln}[-0.0053xln\left(1-xb\right)]}{0.09165}$$

where

$$\begin{array}{c}xb=-19.907\times 0.0336\times albumin+0.0095\times creatinine+0.0195\times glucose\\ +0.0954\times ln\left(CRP\right)-0.0120\times lymphocyte percent+0.0268\\ \begin{array}{c}\times mean cell volume+0.3356\times red blood cell distribution width\\ +0.00188\times alkaline phosphatase+0.0554\times white blood cell count\\ +0.0804\times chronological age\end{array}\end{array}$$

### Calculation of dAge and age ratio

Delta age (dAge) was calculated as the difference between BA and CA (dAge = BA-CA) and reflects the difference in years between both.

The age ratio between BA and CA was calculated as age ratio = BA/CA to reflect the rate of aging of people with diabetes and the selected biomarkers at a specific moment whether a person appears older (value > 1) or younger (< 1) than expected based on their CA. It provides equivalent information as phenotypic age acceleration used in Levine [24, 20].

### Mortality analysis

dAge was calculated at baseline using KDM for individuals recruited to the ACCORD trial with seven biomarkers as specified above. Using SAS v.9.4 (SAS Institute, Cary, NC), we conducted a time-to-event analysis to examine the effects of dAGE, as a continuous predictor, on progression to total mortality in all ACCORD participants without missing biomarker data (*n* = 10,093). Cox-proportional hazards regression models were applied for this analysis, and trial covariates (glycemic, blood pressure, and lipid trial assignments) were included in the model. For illustration purposes, Kaplan–Meier curves were plotted to demonstrate the effects of dAGE above vs. below the median on total mortality in ACCORD.

### Statistical analysis

Statistical analysis was performed using Mann–Whitney-Wilcoxon Test (Wilcoxon rank sum test) in R and Prism.

## Results

### Clinical biomarkers correlate with chronological age in a non-diabetic population

The NHANES 2017–2018 survey was queried for men and women aged 20–80 without a diabetes diagnosis of diabetes and an A1c < 5.7 to exclude undiagnosed prediabetes [17]. A total of 1798 individuals were included in the search of clinical biomarkers that significantly correlated with age. Eight biomarkers (Table 1) were chosen due to the limited availability of biomarkers included in the Joslin T1D and T2D clinical records, and they significantly correlated with CA. The statistical significance of their correlation with CA varied between 1.2 × 10^–10^ and 1.1 × 10^–2^. The *R*^2^ value of creatinine exceeded 0.32 and therefore failed to mitigate the effects of the CA paradox [21]. These biomarkers were chosen to calculate BA in the T2D and T1D cohorts and NHANES 2017–18 prediabetes and diabetes groups. BA was calculated in the ACCORD Trial subjects with the same biomarkers, excluding blood urea nitrogen due to its absence in patient records.

**Fig. 1:**
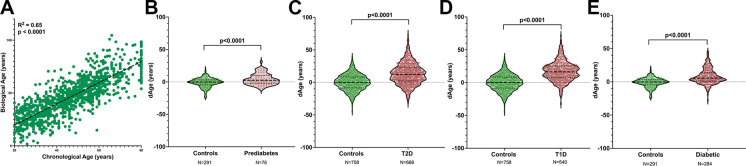
Significant increase in BA in diabetes patients calculated using KDM. **a** Linear correlation between CA and BA for non-diabetic control subject (*n* = 1798); **b** significant increase in BA in subjects with prediabetes (*n* = 66); **c** significant increase in BA in people with T2D (*n* = 686), an average of 12 years greater than controls; **d** BA in people with T1D (*n* = 540) and **e** BA in people with diabetes (T1 and T2D) (*n* = 284)

A strong correlation between CA and BA was observed for people without diabetes (*R*^2^ = 0.65, *p* < 0.0001) (Fig. 1) validating the selected biomarkers and KDM1 as an adequate mathematical model to calculate BA in this population. The strong relationship between CA and BA highlights the convenience of using accessible clinical biomarkers to estimate BA once adequate validation of their correlation with CA has been performed.

### Diabetes mellitus correlated with increased BA

Using KDM1 and the identified biomarkers, BA was calculated in people diagnosed with T2D and their dAge (dAge = BA-CA) compared to CA. On average, dAge of people with T2D in the Joslin cohort was 12.02 years greater than those without diabetes (*p* < 0.0001) (Fig. 1). However, the spread of BA across the population of T2D indicates a variety of additional factors that influence BA alongside a T2D diagnosis. There was a considerable proportion of patients within the diabetes cohort who had a BA lower than their CA indicating that by no means, a diagnosis of T2D inevitably lead to accelerated aging.

To understand whether the increased BA preceded the development of T2D, a population of prediabetes was queried for BA (Fig. 1). Prediabetes was characterized by a 2.69-year significant increase in BA with respect to matched individuals, suggesting that the most significant increase in BA observed in the population with T2D occured after the diagnosis instead of leading up to it. Therefore, accelerated cellular age did not seem to be one of the causal factors of T2D but rather a result of it.

One of the main risk factors to develop T2D is increased age which might suggest that the correlation between the disease and increased BA might be an epiphenomenon brought about by increased CA. We therefore selected a cohort of people with T1D, whose onset is characteristically during childhood or early adolescence [25], and inquired whether in this population a similar change in BA would be observed. A cohort of 540 people with T1D were included and revealed a 16.61-year average increase of BA (*p* < 0.0001) when compared to age-and gender-matched people without diabetes (Fig. 1). Finally, to corroborate these results in a different cohort, BA in NHANES diabetes reported patients was calculated and a significant increase of BA was confirmed when compared to a non-diabetes population (Fig. 1).

These results reveal a significant increase in BA with *diabetes mellitus*, irrespective of age of diagnosis and of the underlying pathophysiology of the disease (metabolic for T2D versus autoimmune for T1D).

### Age ratio correlates with modifiable biomarkers

We have defined age ratio as a surrogate marker of the rate of aging. It determines the correlation between BA and CA (BA/CA) at a given point in time and provides a quantitative value between both ages. An age ratio of 1 indicates a perfect match between BA and CA whereas values > 1 correlate with faster cellular aging and values < 1 correlate with a slower one compared to chronological aging.

**Fig. 2:**
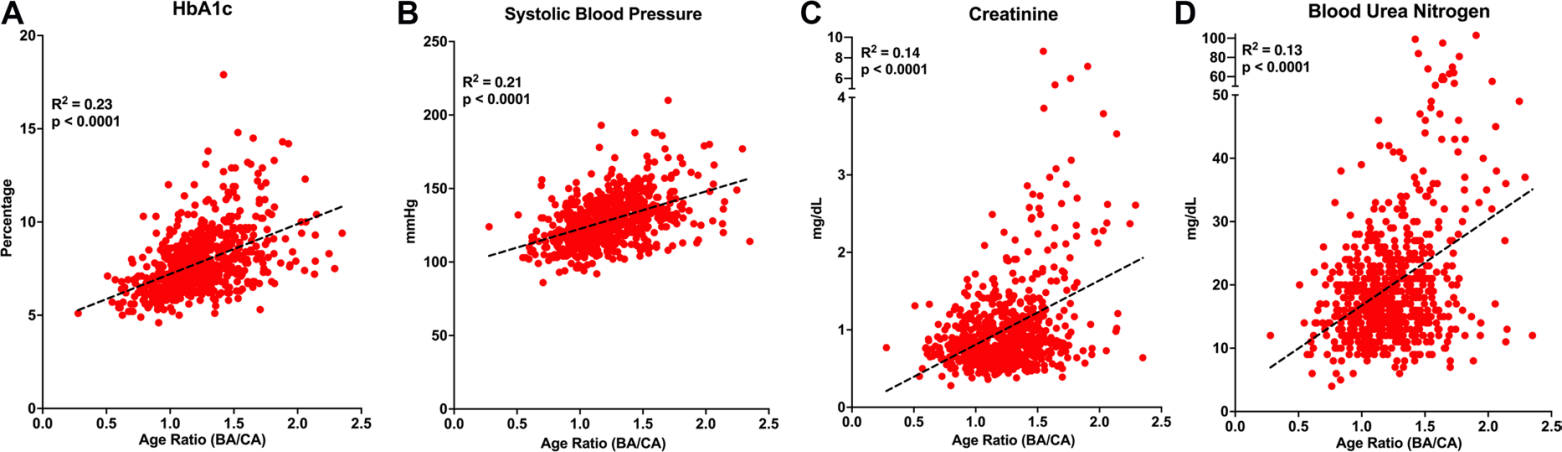
Biomarkers that are strongly correlated to the BA/CA age ratio in a population of T2D. Correlation between age ratio and biomarkers: **a** aA1c (%), **b** systolic blood pressure (mmHg), **c** creatinine (mg/dL), and **d** urea nitrogen (*n* = 686)

To study the contribution of each of the selected biomarkers to BA, linear correlation between biomarkers and the age ratio was calculated for the population with T2D (Fig. 2, Suppl. Figure 1). The two biomarkers with the strongest correlation to age ratio using KDM were A1c (Fig. 2), and systolic blood pressure (Fig. 2); which tightly correlate with metabolic control and cardiovascular health. Other biomarkers that correlate with age ratio using KDM were creatinine and blood urea nitrogen (Fig. 2) reflective of liver and renal function, respectively. Cholesterol, diastolic blood pressure, albumin, and pulse had the weakest correlations to age ratio using KDM (Suppl. Figure 1).

These data underline the importance of proper metabolic control and blood pressure as effective measures to potentially counteract the accelerated BA observed in T2D and is a novel way to interpret and evaluate therapeutic strategies. Interestingly, the correlation between age ratio and individual biomarkers in the population of T1D was much weaker than for T2D, except for A1c, (Suppl. Figure 2). The reason for this dissociation in T1D is unclear and requires further studies.

### Multiple linear regression (MLR) confirms increased BA with Diabetes mellitus

Confirmation of BA results was performed using a complementary mathematical model to estimate its values in the same three populations—NHANES diabetics, prediabetes, and T1D—and compared to a non-diabetes population.

**Fig. 3:**
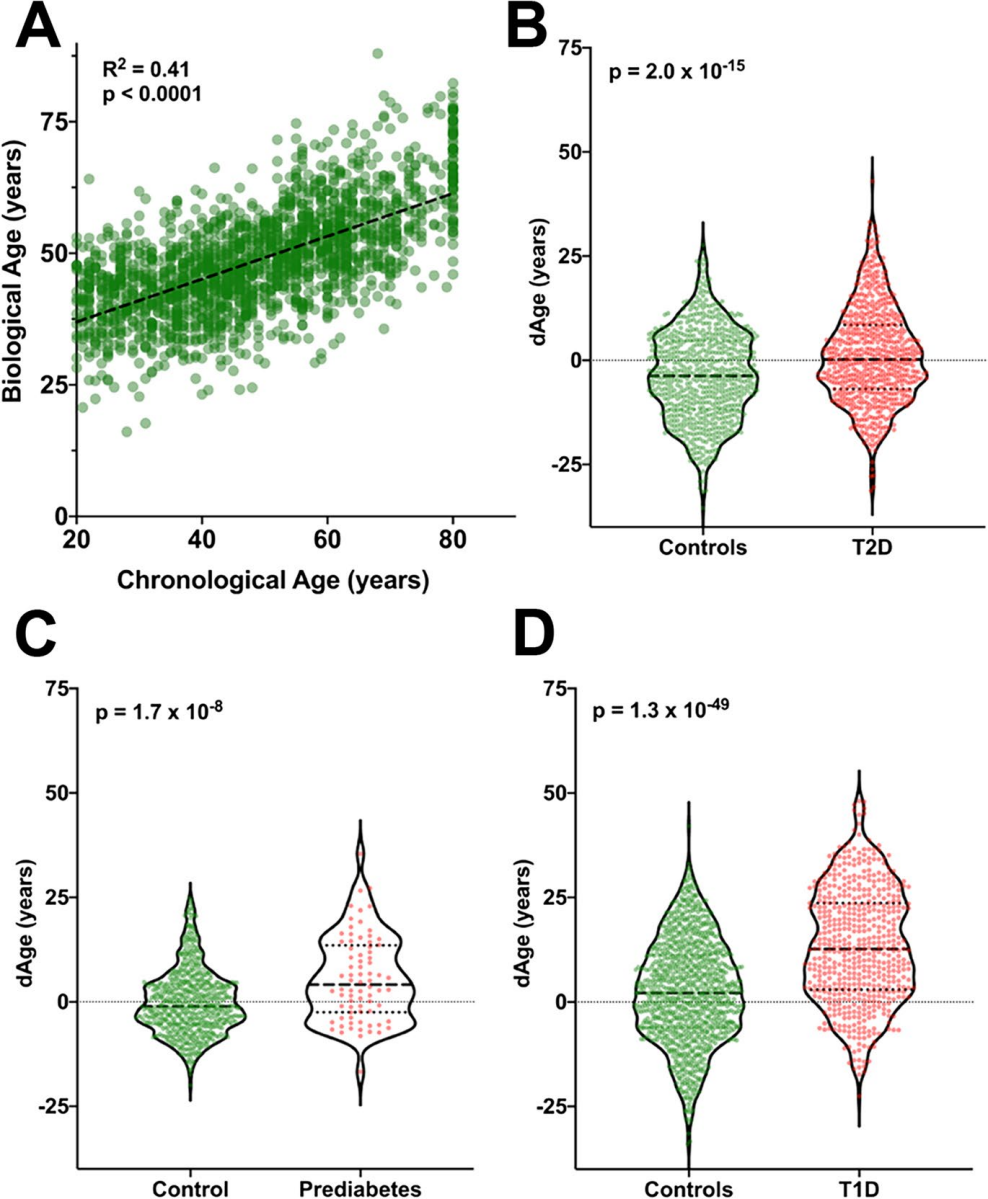
Linear model confirmation of increased dAge in diabetes and prediabetes cohorts. **a** Linear correlation between CA and BA for non-diabetic control subject as calculated with LMR (*n* = 1798). **b** Higher dAge in Joslin T2D cohort (*n* = 686). **c–d** Significant increase in prediabetes (*n* = 76) and Joslin’s T1D cohort (*n* = 540), respectively

The correlation between CA and BA among people without diabetes using MLR (Fig. 3A) (*R*^2^ = 0.41, *p* < 0.0001, slope = 0.19) was not as strong as when KDM1 was used. This is consistent to what has been described in other studies [26, 27], and one of the reasons why KDM1 is preferred to calculate BA. Using MLR, people with T2D displayed a 3.94-year increase in BA compared with age- and gender-matched controls (Fig. 3B) while in prediabetes the average increase was of 5.2 years (Fig. 3C). In the T1D population, BA increase was also confirmed with a value of 10.53 years (Fig. 3D).

When comparing the two mathematical models, KDM-estimated BA tended to show larger differences from CA than MLR, which is consistent with previous findings that MLR estimates of BA regress towards the mean (e.g., people older according to BA are estimated too young) [21]. A main difference between these two methods is that MLR regresses CA on the biomarkers, whereas KDM1 treats CA as an independent variable to find the best model that estimates BA. From a mathematical perspective, the slope of the CA/BA correlation calculated with KDM (Fig. 1) is 1 meaning that in the population without diabetes, a 1-year increase in CA will be reflected as a 1-year increase in BA. However, the slope of the CA/BA correlation calculated with MLR (Fig. 3A) is 0.19 which means that BA calculated with MLR underestimates BA.

### Both PhAge and KDM correlate with increased BA in diabetics and prediabetics

**Fig. 4:**
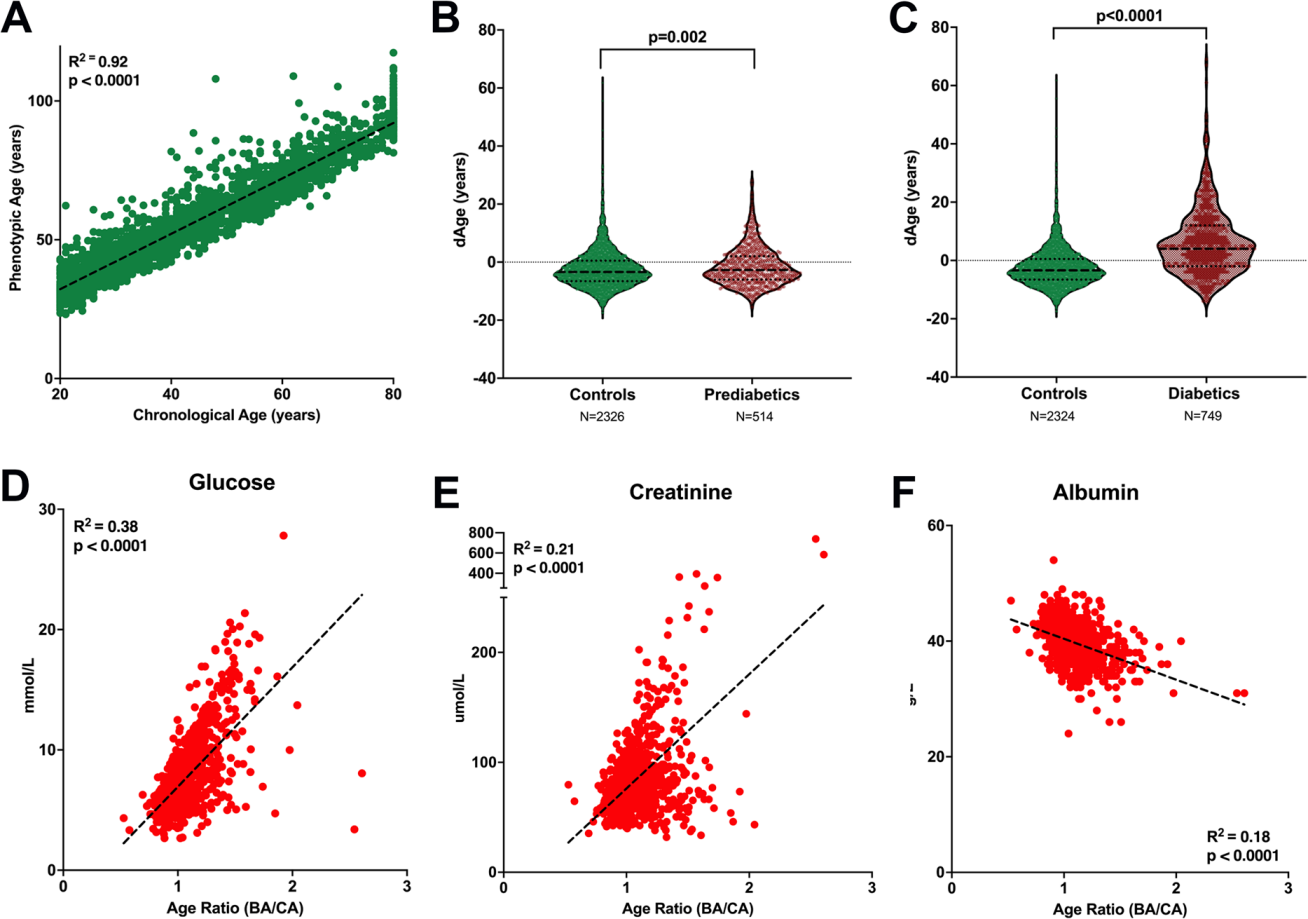
Significant increase in BA in prediabetics and diabetics calculated using PhAge algorithm. **a** Linear correlation between CA and BA for non-diabetic control subjects (*n* = 1798). **b** BA in people with prediabetes (*n* = 514); **c** BA in subjects with diabetes (T1D and T2D (*n* = 749). Correlation between age ratio and biomarkers: **d** glucose (mmol/L), **e** creatinine (umol/L), and **f** albumin (g/L) (*n* = 764)

To understand the importance of biomarkers used to calculate BA, we calculated BA using the PhAge algorithm. A strong correlation between CA and BA was found in non-diabetics using PhAge (*R*^2^ = 0.90, *p* < 0.0001, slope = 0.998) (Fig. 4). PhAge found that prediabetic subjects were 0.89 years greater than those without diabetes (*p* < 0.0001) (Fig. 4), while KDM noted an increase of 2.69 years (*p* < 0.0001). In the NHANES diabetic cohort, PhAge calculated a BA 7.57 years greater than those without diabetes (*p* < 0.0001) (Fig. 4), and KDM calculated a BA 5.73 years greater than nondiabetic subjects (*p* < 0.0001). The three biomarkers with the strongest correlation to age ratio using PhAge were glucose (mmol/L), creatinine (umol/L), and albumin (g/L) (Fig. 4).

Validation of results using two independent models (MLR and PhAge) provided confidence in the selection of biomarkers and in using KDM as a valid model to estimate BA.

### Persistence of effects of diabetes on BA after alternate selection of biomarkers

**Fig. 5:**
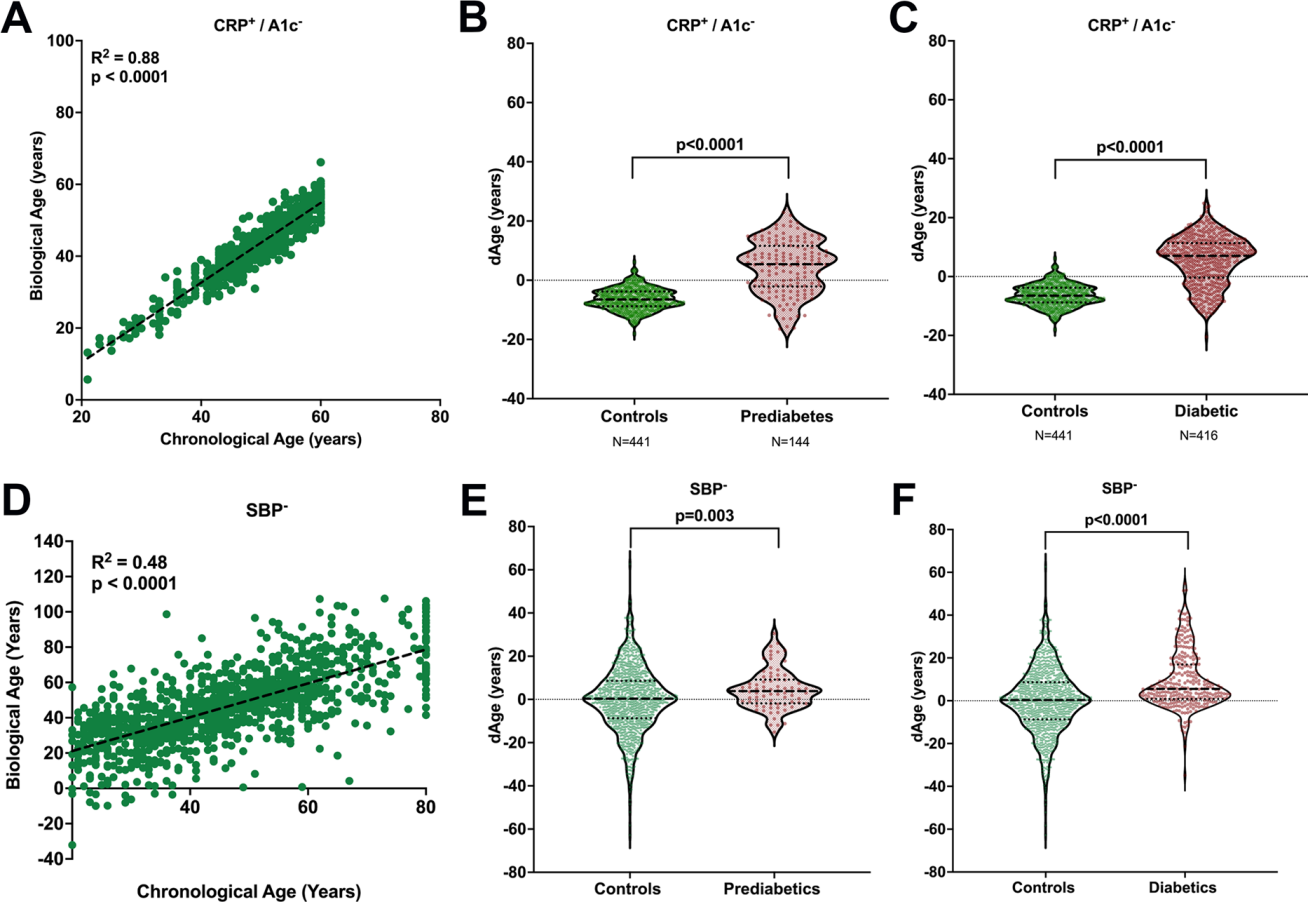
Substitution of A1c for CRP (CRP^+^/A1c^−^) and absence of SBP (SBP^−^) in BA calculations continue to show increased BA in prediabetic and diabetic populations. **a** Linear correlation between CA and BA for non-diabetic control subjects with KDM calculation substituting A1c by CRP. **b** Significant increase in BA in subjects with prediabetes after KDM calculation substituting A1c by CRP. **c** Significant increase in BA in subjects with diabetes after KDM calculation substituting A1c by CRP; **d** linear correlation between CA and BA for non-diabetic control subjects after KDM calculation without SBP; **e** significant increase in BA in subjects with prediabetes after KDM calculation without SBP; **f** significant increase in BA in subjects with diabetes after KDM calculation without SBP

Given the confounding effect that using A1c as a BA biomarker has in prediabetes and diabetes, recalculation of BA using KDM was done by excluding A1c and substituting it with CRP, a commonly used biomarker with a high correlation to CA [2, 20]. A positive and strong correlation between BA and CA persisted (Fig. 5) as did the significant increase of BA in a population with prediabetes (Fig. 5) and diabetes (Fig. 5).

Given the strong correlation that systolic blood pressure (SBP) with BA in people with T2D (Fig. 2), BA was recalculated excluding this marker. A positive correlation between BA and CA also persisted (Fig. 5) as did significant increases in populations with prediabetes (Fig. 5) and diabetes (Fig. 5).

### Effects of BMI and smoking on BA in a population with and without diabetes

**Fig. 6:**
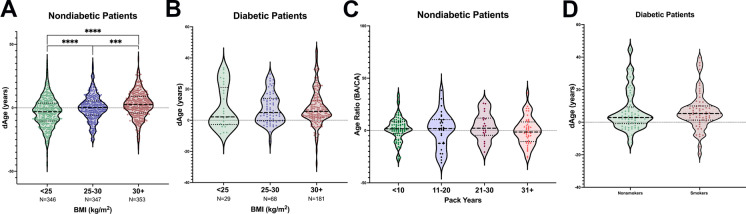
Effects of BMI and smoking status on BA. **a** Significant increase in BA as BMI increases in subjects without diabetes. **b** No significant increase in BA as BMI increases in subjects with diabetes. **c** No significant increase in BA in subjects without diabetes as pack-years increases. **d** No significant difference between BA in subjects with diabetes who were smokers or non-smokers

Given the strong effects that confounding factors such as BMI and smoking can have on clinical biomarkers, BA was calculated across the BMI spectrum and in a smoker and non-smoker population in individuals with and without diabetes. An increase in BMI was associated with increased BA (Fig. 6) in a population without diabetes. However, the significance of this correlation disappeared when diabetes was present (Fig. 6) probably because of the overriding effects of A1c and SBP as previously determined.

Unexpectedly, smoking did not influence the two populations involved (Fig. 6). This is probably secondary to the selection of biomarkers which did not include any lung capacity measures, such as FEV_1_, which have been shown to correlate with BA [2]. This emphasizes the importance of biomarker selection for specific populations.

### Association of dAge and long-term mortality data validates increased BA in diabetes

A limitation of the results presented so far is their cross-sectional nature which impairs validation of results. To address this, BA was calculated in 10,093 individuals enrolled in the ACCORD trial at baseline and correlated with mortality data during the duration of the study.

**Fig. 7:**
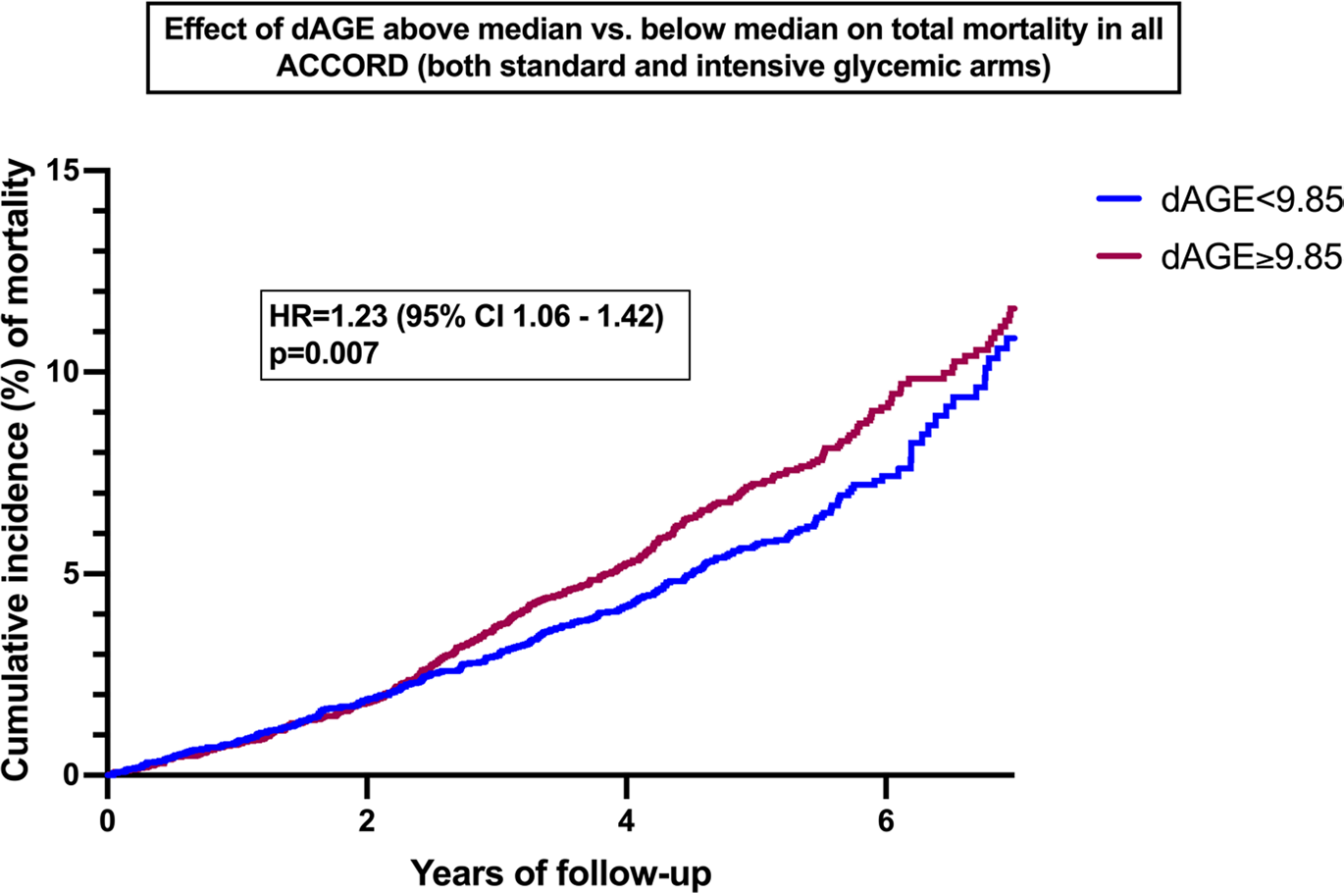
Kaplan-Meier for the effect of dAGE above and below median in ACCORD populations. Effects of dAGE above the median (≥9.85) vs. below the median (<9.85) on incidence of deaths in 10,093 ACCORD participants. The hazard ratio (HR) and p-value are obtained from a cox-proportional hazards model examining the effects of dichotomized dAGE (above vs. below median) on total mortality, adjusted for glycemic, blood pressure and lipid trial assignments

Figure 7 shows the effect of dAge above and below median (9.85 years) in all ACCORD data. An increased HR of 1.23 (95% CI 1.06–1.42) was found when comparing individuals with a dAge equal or greater than 9.85 years with those with a dAge below that number indicating that greater differences between BA and CA carry a greater risk of death. Whereas dAge was calculated at time of enrollment, the difference in mortality becomes apparent after 2 years of follow up and persists thereafter. Interaction analysis showed no change in dAge (*p* = 0.25) after treatment of glycemia between the standard and intensive glycemic arms. Whereas the ACCORD trial followed patients for 7 years, subsequent analysis might find differences secondary to therapeutic interventions after longer follow up as suggested by the legacy effect [28, 29]. The longitudinal nature of this analysis validates the presented results.

In summary, our results reveal increased BA in people with *diabetes mellitus* which in turn suggests accelerated aging. Readily available clinical biomarkers were used favoring the use of KDM1 as a convenient model for BA calculation. The increase in BA seen in both T1D and T2D provide a novel insight into the way tissues behave along a chronological scale, in settings with altered glucose metabolism irrespective of pathophysiological mechanisms. This, along with the strong correlation between A1c and the age ratio, underlines the importance of glucose control in determining aging at a cellular level.

## Discussion

The correlation between T2D and age is complex, and studying the disease from an aging point of view can provide novel mechanisms and therapeutic targets. In this study, using the KDM1 model to calculate BA in people with T1D and T2D, we correlated for the first time at an epidemiological level an increased BA with the diagnosis of T2D and preceded by prediabetes. The increased BA also observed in T1D, which is not age dependent, suggests that age acceleration is dependent on altered glucose metabolism rather than on peripheral insulin resistance or cell autonomous mechanisms that characterize T2D.

Understanding the increased BA with diabetes and the subsequent accelerated aging is critical to further our understanding of the biology of aging in health and disease. Multiple studies support the concept of accelerated aging in diabetes: increased telomere shortening and mitochondrial DNA depletion in patients with T2D [30] and accelerated aging of human collagen in juvenile *diabetes mellitus* as determined experimentally by enzymatic digestion [31]. Particularly, cellular senescence has also been reported to be increased in the setting of glucose metabolism dysregulation. Hyperglycemia accelerates vascular aging by inducing senescence in endothelial cells, a process suggested to be mediated by ASK1 [15] and p38MAPK [23]. Fibroblasts from skin biopsies underwent cellular senescence earlier if the donor was diagnosed with T1D or had a strong family history of T2D [23]. We have shown that β-cell aging and senescence is accelerated in islets from donors with T2D and higher body mass indices, a potential indirect marker of insulin resistance [32, 33].

The ability to measure changes in the rate of aging using readily available clinical biomarkers represents a powerful tool to track this phenomenon and to evaluate clinical interventions. Since the KDM1 method does not limit which biomarkers can be included in the calculation as long as they have a significant relationship with CA, BA research can be done to study more markers depending on availability. All of the 8 biomarkers we used (albumin, creatinine, systolic blood pressure, glycated hemoglobin, diastolic blood pressure, pulse, cholesterol, and blood urea nitrogen) overlap with previous reports of biological age analysis by KDM as reported by Levine et al. [2]. However, that list is more extensive and includes additional biomarkers such as forced expiratory volume, serum alkaline phosphatase, C-reactive protein, and cytomegalovirus optical density, which we were unable to include in our biomarkers list, since they were not available in our data set. It is interesting to note that our data did show that pulse was associated with CA in our non-diabetic controls, whereas the Levine M. E. study did not. Another interesting difference is the correlation between age and A1c which is lower in our study. We believe this is explained by our exclusion of individuals with an A1c equal or greater to 5.7%, defined as prediabetes by the American Diabetes Association (ADA).

Further studies of telomere shortening, DNA methylation, and mitochondrial DNA depletion in populations with diabetes would further our understanding of accelerated aging and would contribute to directly measure the level and extent of damage accumulation in the body in health and disease. Identification of the proper DNAm profile would be crucial in these studies since correlations between KDM BA and some DNAm clocks have been shown to be quite low [34], whereas the use of machine learning analysis of pace of aging found to have a better correlation with increased decline of physical function, cognitive function and subjective signs of aging over a 7-year period [8]. A recent paper [35] demonstrates that age-related epigenetic changes can be reverted to DNA methylation profiles characteristics of younger ages, speaking to the concept of BA being a dynamic, rather than a static, parameter.

Many of the biomarkers that strongly correlate to BA are known to be altered in T2D and its complications, implying that disease severity is one of the determinants of BA. For example, evidence has shown that premature aging in T2D leads to an increased risk of cardiomyopathy [27] and our study found a high correlation between age ratio and systolic blood pressure, which promotes myocardial remodeling and is a risk factor in the development of cardiomyopathy [28, 29]. To further evaluate the effect of disease-dependent biomarkers such as A1c, we performed KDM analysis in prediabetes and diabetes population excluding A1c and including CRP. The increase in BA persisted suggesting that there are cell autonomous mechanisms that are altered in diabetes and are not solely dependent on blood glucose levels and lead to accelerated aging.

Additionally, given the strong correlation between biomarkers such as systolic blood pressure and A1c to BA and the existence of effective clinical interventions that can modify them, the concept of BA becomes potentially dynamic and modifiable. Other studies have shown that BA is modifiable such as the CALERIE intervention where participants randomized to caloric restriction revealed decrease in the rate of biological aging [36]. However, interaction analysis showed no change in BA due to therapeutic intervention in the ACCORD study analyzed in the current paper. This could be secondary to the legacy effect showing lasting changes (up to 10 years) due to changes in blood glucose levels [28, 29]. Since this ACCORD cohort was followed for an average of 5–7 years, perhaps further follow-up or inclusion of additional biomarkers might reveal BA changes after glucose and blood pressure interventions.

Based on these concepts, we propose BA can be used as an additional clinical outcome to track the evolution of individual patients, their lifestyle, and influence of pharmacological interventions, in such a way that some biological processes of aging may be slowed as discussed in the geroscience hypothesis [37].

### Significance and limitations of the study

Most patients with T2D are above the fifth decade of life, suggesting a correlation between cellular age and diabetes. This paper identified an increase in biological age (a reflection of aging at a cellular level) compared to chronological age (defined by time since birth) in people with *diabetes mellitus*. The significance of these findings is (1) aging at a cellular level is accelerated in diabetes and (2) biological age, which reflects the rate of aging, can be calculated using readily available clinical biomarkers and can guide interventions to improve health span and lifespan.

Although there was a very strong and consistent relationship between accelerated aging and diabetes, there are limitations in this study that need to be addressed.

First, the biomarkers used were limited given the constraints of NHANES, ACCORD, and Joslin Diabetes Center patient data.

Second, the Joslin cohorts are not a nationally representative sample. There are several genetic and environmental factors that affect the aging process that are important to consider. Thus, we found a relationship between aging and diabetes here, but the model is yet to be extended to human aging in general.

Third, the NHANES cohort differentiates patients based on their response to the question asking whether they have been diagnosed with diabetes or prediabetes. Therefore, type 1 and type 2 diabetes cannot be differentiated in this cohort and the data relies on the subjective response of the participant, allowing for the possibility of information bias.

In conclusion, the development and study of aging beyond traditional CA constraints is vital to understand the aging process and how to address conditions that affect it. Moving forward, integrating our theoretical and cellular understanding of aging with environmental, behavioral, and heritable factors is necessary to facilitate future development in the field of aging and diabetes research.

## Supplementary Information

Below is the link to the electronic supplementary material.

Supplementary file1 (DOCX 443 KB)
