# Supplementary material for: Diabetes mellitus correlates with increased biological age as indicated by clinical biomarkers
Source: GeroScience. 2021 Nov 12;44(1):415–27. doi: 10.1007/s11357-021-00469-0 (PMC8589453; doi:10.1007/s11357-021-00469-0)
Supplement: Supplementary file 1 — Supplementary file1 (DOCX 443 KB) [file 11357_2021_469_MOESM1_ESM.docx]

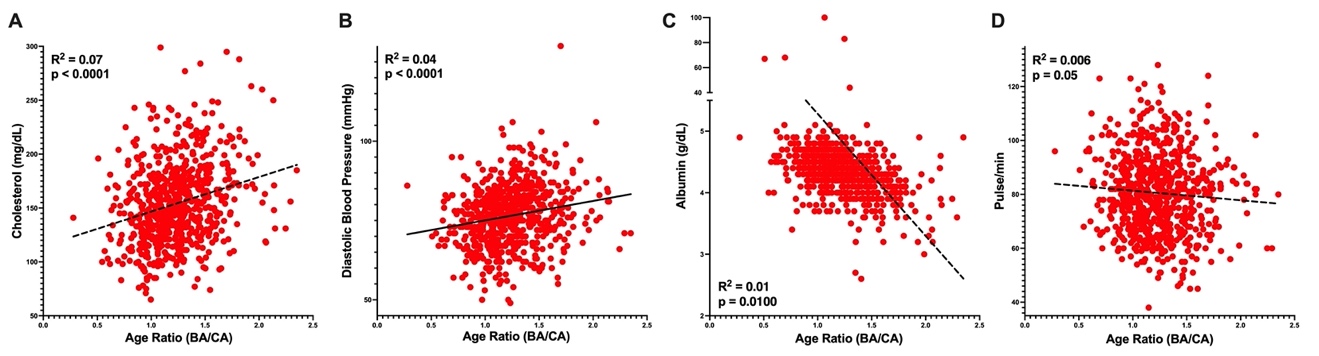


**Supplementary Figure 1. Biomarkers weakly correlated to the BA/CA ratio in the T2D population.**

(**a**) Cholesterol (mg/dL); (**b**) Diastolic blood pressure (mmHg); (**c**) Albumin (g/dL); (**d**) Pulse (/min) are weakly correlated with the rate of aging (n=686).


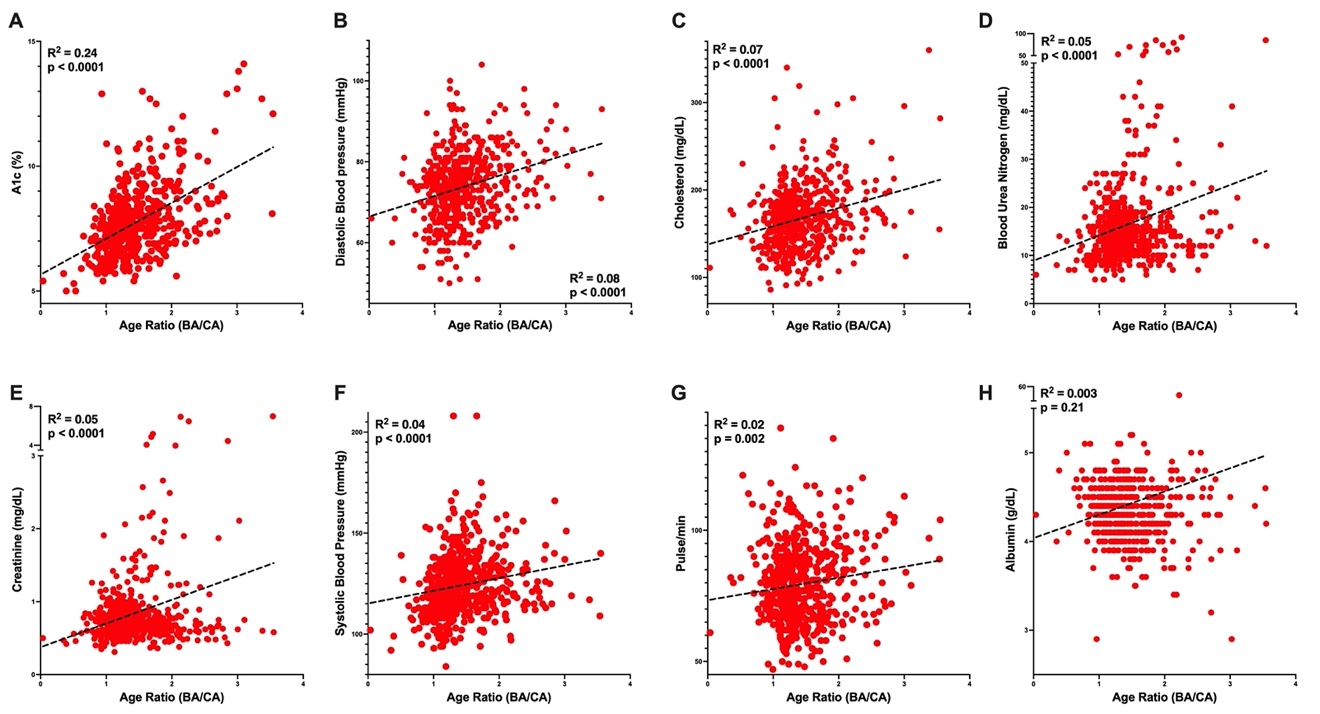


**Supplementary Figure 2. Biomarkers correlation to the BA/CA ration in a population of T1D.**

(**a**) A1c (%); (**b**) Diastolic blood pressure (mmHg); (**c**) Total Cholesterol (mg/dL); (**d**) Blood Urea Nitrogen (mg/dL); (**e**) Creatinine (mg/dL); (**f**) Systolic blood pressure (mmHg); (**g**) Pulse/min; (**h**) Albumin (g/dL) (n=540).

**Suppl. Table 1**. Summary of age in matched data for Joslin Patients and NHANES controls

| Cohort | Gender | n | Age Mean | Age Standard Deviation |
| --- | --- | --- | --- | --- |
| Control Test | Male | 372 | 45.2 | 17.2 |
| Control Test | Female | 386 | 46.7 | 16.4 |
| T1D | Male | 229 | 44.9 | 15.1 |
| T1D | Female | 311 | 43.6 | 14.6 |
| T2D | Male | 388 | 58.5 | 9.2 |
| T2D | Female | 298 | 57.4 | 10.4 |
| Control Train | Male | 696 | 45.7 | 17.0 |
| Control Train | Female | 821 | 46.1 | 16.6 |

**Suppl. Table 2.** Summary of age in matched data for people with diabetes, prediabetes and age matched controls. NHANES 2017-2018

| Cohort | Gender | n | Age Mean | Age Standard Deviation |
| --- | --- | --- | --- | --- |
| Nondiabetic (test) | Male | 137 | 48.5 | 8.1 |
| Nondiabetic (test) | Female | 154 | 49.4 | 8.0 |
| Prediabetic | Male | 36 | 47.1 | 12.1 |
| Prediabetic | Female | 40 | 46.4 | 10.3 |
| Diabetic | Male | 147 | 50.7 | 7.8 |
| Diabetic | Female | 137 | 49.2 | 8.7 |
| Nondiabetic (train) | Male | 270 | 48.3 | 9.0 |
| Nondiabetic (train) | Female | 312 | 47.7 | 8.6 |
